# Gingival Pigmentation Features in Correlation with Tooth and Skin Shades: A Cross-Sectional Study in a Saudi Population

**DOI:** 10.3290/j.ohpd.b4347777

**Published:** 2023-08-30

**Authors:** Amani Mirdad, Mayson Alqarni, Areej Bukhari, Razan Alaqeely

**Affiliations:** a Assistant Professor, College of Dentistry, King Saud University, Riyadh, Saudi Arabia. Conceptualization, study idea, wrote the manuscript.; b Postgraduate in Periodontics, College of Dentistry, King Saud University, Riyadh, Saudi Arabia. Collected data, examined patients.; c Dental Intern, College of Dentistry, King Saud University, Riyadh, Saudi Arabia. Collected data, examined patients.; d Assistant Professor, College of Dentistry, King Saud University, Riyadh, Saudi Arabia. Study design, data analysis, wrote the manuscript.

**Keywords:** gingival pigmentation, hyperpigmentation, prevalence, skin colour, tooth shade

## Abstract

**Purpose:**

The present study aimed to observe the anatomical distribution of gingival melanin pigmentation and evaluate its intensity and extent in different age groups and in correlation with skin and tooth shades.

**Materials and Methods:**

The participants of this study were 391 patients attending the Dental University Hospital. The presence of gingival pigmentation was assessed using De Krom’s Oral Pigmentation Chart and its intensity was assessed using the Dummett-Gupta Oral Pigmentation Index. Skin colour and tooth shade were measured using the Fitzpatrick scale and the VITA classical shade guide, respectively. Statistical analyses included descriptive statistics and Pearson’s Χ^2^ test for the association between the study variables.

**Results:**

The prevalence of gingival pigmentation among the sample size was 74.4%, and pigmentations were present on both arches in 57.6% (n = 224) of the participants. The extent (category 1) was highest when pigmentation was evident in both arches, with category 4 being the least extent. Age and sex did not show a correlation with gingival pigmentation. Gingival pigmentation intensity was mild when pigments were present in one arch (p < 0.00), whereas it was heavy when both arches presented with gingival pigmentation. Medium brown colour and tooth shade A1 were the most common among participants with gingival pigmentation (p < 0.00). The association between gingival pigmentation intensity and extent in relation to skin colour was statistically significant (p < 0.00), as was tooth shade (p < 0.05).

**Conclusions:**

Gingival pigmentation is highly prevalent in the Saudi population, with different severity and extent levels. The effect of gingival pigmentation on smile and overall facial aesthetics should be considered when providing dental and cosmetic treatments.

Oral hyperpigmentation is characterised by an increase in oral pigmentation beyond normal levels.^20^ While it is a relatively common condition that may involve any part of the oral cavity,^7^ it mostly affects gingival tissues.^23^ It is more common in dark-skinned populations.^3,9^ In some populations, it is considered a genetic trait; therefore, it is termed physiological (racial) pigmentation.^4,6,21^ It is caused by several physiological and pathological factors. However, excessive deposition of melanin by melanocytes is mostly caused by physiological factors.^9^

Healthy gingival colour varies from pale and coral pink in Caucasians to brownish and blue-blackish in Asian or African populations.^19^ Some studies have reported a strong relationship between facial skin tone and the severity of gingival pigmentation, concluding that people with fair skin tones tend to have mild gingival pigmentation, whereas those with darker skin tones tend to have moderate to severe gingival pigmentations.^14,18,22^

In addition, various studies have suggested a relationship between facial skin complexion and gingival tissue pigmentation.^12^ Race is another variable that affects the intensity and distribution of pigmentation patterns.^13^ The population of Saudi Arabia has diverse skin-colour complexions, social factors, and ethnic groups.

The present study aimed to assess the anatomic distribution of physiological gingival melanin pigmentation and to evaluate the correlation between skin colour and tooth shade.

## Materials and Methods

The protocol of this cross-sectional study was approved by the Ethics Committee of King Saud University (No. 21-0232). The participants were recruited from patients visiting the King Saud University Dental Hospital in Riyadh, Saudi Arabia. Informed consent was obtained from all participants or parents of those participants below 16 years of age.

The inclusion criteria were as follows: Saudi citizen, ten years of age or older, and had not undergone periodontal treatment. The exclusion criteria were as follows: any systemic disorders that influence gingival pigmentation (including diabetes mellitus, Peutz-Jeghers syndrome, Addison’s disease, Albright syndrome, melanoma, periodontitis, or any gingival pathology-induced colour changes); habits such as smoking, quid chewing, or gingival placement of other substances; and restorations, prosthetic crowns, or veneers on the maxillary central incisors.

Female participants were asked to wipe off their lipstick or lip gloss, if any, before the shade evaluation procedure.

**Fig 1:**
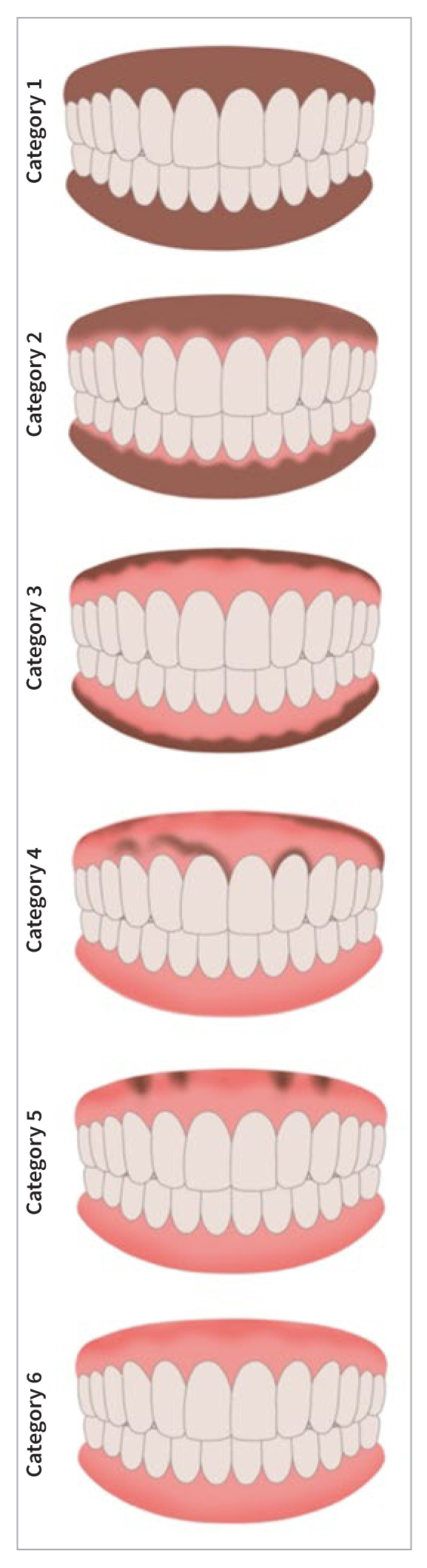
De Krom’s oral pigmentation chart. *Category 1:* Broad zone of pigmentation surrounds the dentition. The zone is symmetrical and the colour is uniform. *Category 2:* Narrow zone of nonpigmented gingiva surrounds the teeth (i.e. the free marginal gingiva is pink). The zone is symmetrical and the colour is uniform. *Category 3:* Mucosa is completely pink, except for a thick or thin line of pigmentation at the level of mucogingival border. The pigmented zone is symmetrical and the colour is uniform. *Category 4:* Pigmentation is spread over the mucosa. It is asymmetrical, patchy, and irregular. *Category 5:* Mucosa is completely pink, except for several symmetric ‘islands’ of pigmentation between the anterior teeth. *Category 6:* Mucosa is completely pink, and there are no signs of melanin pigmentation.

**Fig 2:**
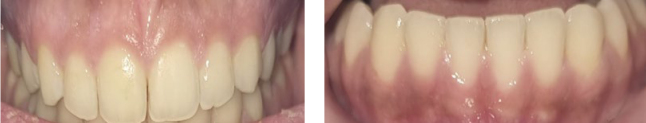
An upper arch presenting with Score 2 in intensity and Category 5 in extent (left), while the lower arch has Score 3 in intensity and Category 3 in extent (right).

The prevalence of gingival pigmentation was determined using the Dummett-Gupta Oral Pigmentation Index^7^ as follows: no gingival pigmentation = 0, mild gingival pigmentation = 1, moderate gingival pigmentation = 2, and heavy gingival pigmentation = 3. In participants scoring 1 or more (i.e. the presence of gingival pigments), the extent and distribution were assessed using De Krom’s Oral Pigmentation Chart,^2^ as shown in Fig 1. Figure 2 shows an example of this.

The facial skin complexion was recorded using the Fitzpatrick skin typing test.^10^ The scale includes complexions ranging from light to dark brown. Skin colour was selected from the malar region of the face.

Regarding tooth shade, the participants were placed in an upright position, and their gingival tissue was dried prior to shade selection under daylight. The VITA classical shade system (A1–D4, Vita Zahnfabrik; Bad Säckingen, Germany) was used against the middle one-third of the labial surface of the permanent maxillary central incisors to determine shade type.

Sample size was determined using G*power sostware. Data were analysed using the SPSS software (version 26.0, IBM; Armonk, NY, USA). Descriptive statistics (frequencies and percentages) were used to describe the categorical study and outcome variables. Pearson’s Χ^2^ test was used to assess the association between the prevalence of gingival pigmentation and the demographic and clinical characteristics of gingival pigmentation. A p-value ≤ 0.05 was used to report the statistical significance of the results.

## Results

### Demographic Characteristics

The total number of participants was 389 with nearly equal male and female participation rates (47.6% male and 52.4% female). Approximately one-third of the participants (32.4%) were aged between 21–40 years.

### Gingival Pigmentation

**Fig 3:**
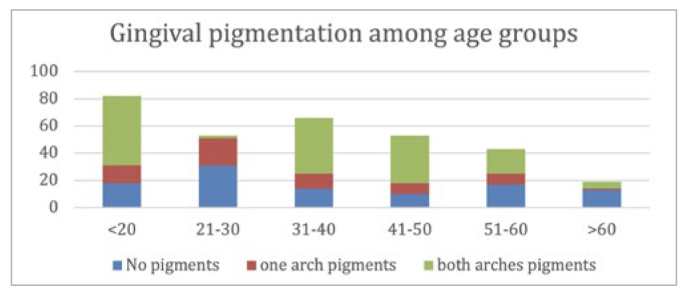
Distribution of gingival pigmentation in relation to age group.

Among the study participants, the prevalence of gingival pigmentation was 74% (n = 288), with no statistically significant difference between male and female participants (72.6% and 74.15%, respectively). Despite the high number of participants younger than 20 years of age, there was no statistically significant difference in the presence of gingival pigmentation within the other age groups (Fig 3). In each age group, a strong statistical significance was observed for the presence of gingival pigmentation (p < 0.01), especially when both arches had pigmentation (p < 0.00).

### Distribution, Extent, and Intensity of the Gingival Pigmentation

**Fig 4:**
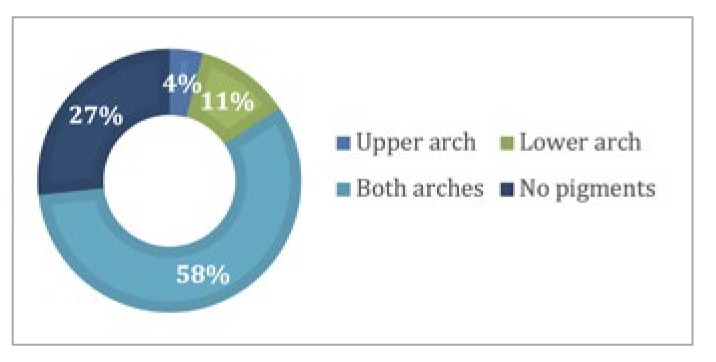
Distribution of gingival pigmentation according to presence in arches.

Figure 4 shows the distribution of gingival pigmentation by arch. In 57.6% (n = 224) of participants, pigmented gingival tissues were present in both arches, followed by only the lower arch (11.6%), and only the upper arch (4.1%). The difference in distribution was statistically significant (p = 0.037).

The extent of gingival pigmentation varied among the participants. Among participants with only upper-arch pigmentation, six out of 17 participants (35.3%) had Category 2, followed by Category 5 (n = 4, 23.5%). Among participants with only lower-arch pigmentation, Category 5 was dominant in 16 participants (37.2%), followed by Category 2 (n = 11, 25.6%) and 3 (n = 10, 22.7%). The difference was statistically significant (upper: p < 0.01, lower p < 0.00). Among participants with gingival pigmentation in both arches, 123 (55%) had the same extent in both arches, and 101 (45%) had different distribution (Table 1). Overall, Category 1 was dominant when both arches had the same level of pigmentation (p < 0.000). Categories 3 and 5 were dominant when arches had differences in extent categories (p < 0.00). Category 4 was the least common among the different distribution patterns (p < 0.03).

**Table 1:** The extent and intensity of gingival pigmentation according to its occurrence per arch

Arch	Extent of GP by category						Intensity of GP by score			
	1	2	3	4	5	6	0	1	2	3
Upper only	2	6	2	2	4	0	0	10	2	4
Lower only	4	11	10	3	16	0	0	24	8	11
Both arches (similar)	39	21	28	19	16		0	64	48	75
Both arches (different)	34	35	55	28	50			32	30	12

GP: gingival pigmentation.

The intensity of gingival pigmentation was measured using the Dummett-Gupta oral pigmentation index; more than half of the participants with only upper or lower arch pigments scored 2 (mild clinical pigmentation) (Table 1). The difference was statistically significant (upper arch only, p < 0.000; lower arch only, p < 0.001). When both arches had pigments, a similar score was obtained for 187 participants. Heavy gingival pigment (Score 4) was the most common (40%), followed by Score 2 (34%), with no statistically significant difference. Twenty-five percent showed moderate gingival pigments, and the difference was statistically significant compared to the other scores (p = 0.03). A small number of participants (n = 36) had different intensity scores between the upper and lower arches; Scores 2 and 3 were dominant. When the intensity score was different between the upper and lower arches, approximately 66% of the participants had darker pigments (Scores 3 and 4) in the lower arch. This difference was statistically significant (p < 0.00).

### Facial Skin Colour

**Fig 5:**
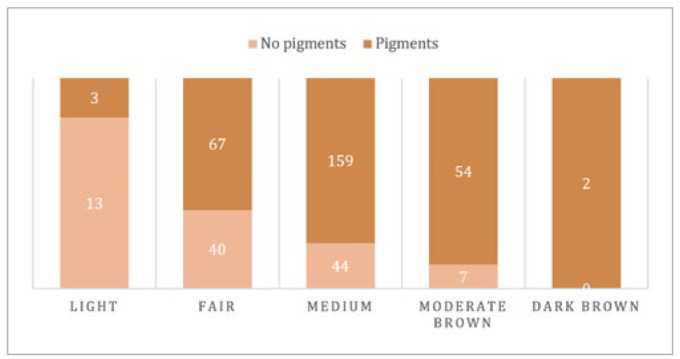
Skin colour distribution according to presence of gingival pigmentation.

Figure 5 shows the frequencies of different skin-colour shades. Approximately half of the participants presented with medium brown skin and had the highest gingival pigmentation prevalence (p = 0.001). Only two people presented with dark brown skin, and both had gingival pigments in both arches. The presence of gingival pigments was statistically significant for all skin colours (p < 0.000), except for light skin. Three of 16 people with light skin colour had gingival pigmentation (18.75%). Gingival pigmentation extent had higher values (Categories 1 and 3) in participants with fair and moderate brown skin (p = 0.008 and 0.001, respectively). For gingival pigmentation intensity, heavy pigments (Score 4) were statistically significant for medium, moderate, and dark brown skin (p = 0.00). Fair skin had a score of 2 when gingival pigmentation was detected. This difference was statistically significant (p = 0.00).

The association between gingival pigments intensity and the extent of skin colour was statistically significant (p < 0.000).

The tooth shade was selected using the VITA classical shade system. This system groups the shades according to hue: A1–A4 are reddish-brown, B1–B4 are reddish-yellow, C1–C4 are grays and D2–D4 are reddish-gray.

**Fig 6:**
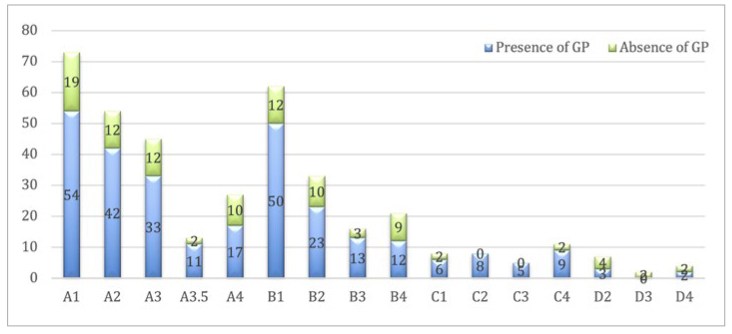
Distribution of tooth shade among participants.

The shading ranged from A1 to D4 (Fig 6). Hue A was the most common among the participants, and Hue D was the least common (p < 0.000). The most common shades among participants with gingival pigmentation were Shade A1 (n = 54) and B1 (n = 50), followed by A2 (n = 42), with no statistically significant difference (p = 0.06). The same pattern of common tooth shades was observed in participants with no gingival pigmentation (A1 = 19, B1 = 12, A2 = 12). A small statistically significant association was observed between tooth shade and the presence of gingival pigmentation (p = 0.05).

**Fig 7:**
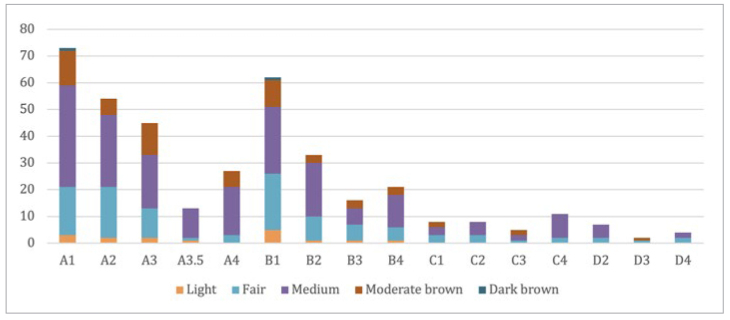
Distribution of tooth shade among different skin colours.

Regarding the different intensities and extents of gingival pigments, only the extent in the lower arch was slightly statistically significant in relation to tooth shade (p = 0.007). Figure 7 shows the distribution of facial skin colour. No statistical difference was observed in any shade among the different skin colours (p = 0.9). However, for medium skin colour, tooth shade A1 was more pronounced than the other shades.

## Discussion

Gingival colour alteration in the form of gingival pigmentation is one of the mucogingival conditions found as a variation of the normal gingival colour. Pigmentation can occur naturally, due to genetics (racial pigmentation), or be caused by factors such as medications or smoking. These pigmentations vary in location and quantity, owing to several factors, including melanocyte count, keratinisation degree, and vascularisation.^1,5,8^ Identifying the prevalence of gingival pigmentation aids in choosing suitable shades for restorative and prosthetic purposes. The need for these increases when the harmony between white and pink aesthetics is of concern (e.g. in patients with a high lip line or a hyperactive upper lip). This will also aid in providing better aesthetic results according to the patient’s age, sex, and skin colour. The aim of this study was to assess gingival pigmentation and its correlation with age, sex, and skin colour in the Saudi population.

In this study, most participants had gingival pigmentation in both the upper and lower arches. This high percentage may be attributed to ethnic or genetic factors. This was in accordance with a study conducted in the Aljouf region of Saudi Arabia, in which gingival pigmentation was prevalent among more than 60% of the participants.^11^

Age was a factor in the higher prevalence of gingival pigmentation in the older age groups. Lungo et al^15^ agreed with our finding even though their sample size included only Black people. The authors evaluated the distribution, intensity, and extent of 70 dark-skinned individuals of known age and sex.^15^ In another study conducted on paediatric patients,^16^ age was not a factor. However, this could be explained by the fact that, in our study, differences in gingival pigmentation was examined in individuals older than 20 years, which the previous study did not.^16^ No known study has found a correlation between sex and gingival pigmentation.

The intensity of melanin pigmentation was not statistically significantly different among the participants, but the extent of the pigmentation was, with the highest scores being 3 in the upper arch and 4 in the lower arch. This finding is in agreement with that of Lungo et al;^15^ in contrast to the extent and distribution, the intensity was not statistically significant among their study sample.

Skin colour can be used to determine the intensity of gingival melanin pigmentation. In our study, most participants had medium-brown skin, followed by fair skin. The presence of pigments results in darker skin. In addition, high scores of gingival pigment intensity and extent were correlated with dark skin, which is in line with previous studies.^15^ Ponnaiyan et al^17^ found that the correlation between skin colour and the intensity of pigmentation was statistically significant in South Indians; heavy gingival pigmentation was observed among dark-skinned participants.

Tooth shade was not correlated with gingival pigmentation among the participants in this study. However, with increasing values, the prevalence of gingival pigmentation was high. This is in agreement with Ghani et al,^12^ who found that darker shades of teeth were more common in participants with gingival pigmentations.

Taking into consideration the skin/teeth/gingival pigmentation complex, this study might give insight into the Saudi population, the majority of whom showed medium skin colour along with a moderate amount of gingival pigmentation and relatively light toothshades. The complex can be considered balanced. The present study emphasises the importance of considering the nature of the Saudi population, where gingival pigmentations are highly prevalent. The complexity of skin colour, tooth shade, and gingival pigmentation must be carefully analysed and considered when planning cosmetic dental and perioral procedures.

The present study has a limitation. It was conducted during the coronavirus 2019 pandemic, which negatively affected the progress of the study and limited data collection. Another factor that would strengthen the results is the personal awareness of the populations’ own gingival pigmentation. This addition might allow a better understanding of peoples’ needs when they seek cosmetic dental treatment.

## Conclusion

Gingival pigmentation is highly prevalent in the Saudi population, with different levels of severity and extent. Gingival pigmentations are mostly related to skin tone rather than tooth shade. The effect of gingival pigmentation on the smile as well as overall facial aesthetics should be considered when providing dental treatment, especially in the anterior teeth and in individuals with a high lip line.
